# Investigation of the wear resistance of different artificial teeth materials in removable dentures

**DOI:** 10.6026/9732063002001159

**Published:** 2024-09-30

**Authors:** Atul Bhandari, Sneha Saraf, Nabarun Chakraborty, Arpita Srivastava, Ishan Roy Choudhury, Kritika Rajan

**Affiliations:** 1 Department of Prosthodontics, Crown and Bridge, RKDF Dental College, Bhopal, M.P., India; 2 Department of Prosthodontics, Pacific Dental College and Research Center, Udaipur, Rajasthan, India; 3 Department of Prosthodontics and Crown and bridge, Dr.R.Ahmed Dental College, Kolkata, India; 4 Department of Oral Medicine and Radiology, Government College of Dentistry, Indore, M.P., India

**Keywords:** Wear resistance, removable dentures, acrylic resin, composite resin

## Abstract

This study aimed to evaluate and compare the wear characteristics of acrylic resins, composite resins and ceramic materials used in removable dentures. A total of 88 samples (n = 88) were subjected to simulated chewing cycles using a wear-testing apparatus. The wear depths were measured using profilometry and statistical analyses were performed to assess the differences among the materials. Ceramics exhibited superior wear resistance compared to acrylic and composite resins in removable dentures. These findings highlight the importance of material selection for the optimization of denture longevity and patient satisfaction.

## Background:

The selection of materials for removable dentures is critical for ensuring their long-term durability and functional performance in clinical practice. Among other mechanical properties, wear resistance plays a pivotal role in determining the suitability and longevity of denture materials. Removable dentures serve as essential prosthetic devices for patients with missing teeth, restoring oral function, aesthetics, and overall quality of life [1]. The materials used in denture fabrication must withstand the dynamic forces of mastication and resist wear to maintain their structural integrity and functional stability over time [2]. Acrylic resin has been the material of choice for denture base construction because of its ease of manipulation, cost-effectiveness, and acceptable aesthetic properties [3]. However, acrylic resins exhibit limitations in terms of wear resistance, susceptibility to surface degradation and potential for microbial adhesion, leading to concerns regarding long-term durability and patient comfort [4]. Composite resins represent a new generation of denture materials that offer improved mechanical properties and aesthetic outcomes compared to traditional acrylic resins [5]. These materials combine a resin matrix with reinforced fillers, such as glass or ceramic particles, to enhance strength, wear resistance, and color stability [6]. Composite resins have gained popularity in denture prosthetics owing to their ability to mimic natural tooth appearance and provide satisfactory functional performance [7]. Nonetheless, their wear characteristics and longevity in clinical settings require thorough evaluation to optimize the material selection and patient outcomes [8]. Ceramic materials, particularly high-strength ceramics such as zirconia and alumina, have revolutionized dentistry because of their superior mechanical properties and wear resistance compared with resin-based materials [9]. Ceramics are well known for their high hardness, biocompatibility, and minimal abrasive wear, making them ideal for applications demanding robust material performance, such as fixed dental prostheses and implant-supported restorations [10]. In removable dentures, ceramics present a compelling option for patients seeking durable and aesthetically pleasing prosthetic solutions with enhanced wear resistance and longevity [11]. Despite advancements in denture materials, there remains a need to systematically evaluate and compare the wear resistance of acrylic resin, composite resin and ceramics in removable dentures. The existing literature provides valuable insights into the mechanical properties and clinical performance of these materials [12, 13]. However, direct comparative studies focusing on wear characteristics under simulated chewing conditions are limited [14]. Such studies are essential for guiding evidence-based decision-making in material selection, optimizing denture design and improving patient satisfaction and clinical outcomes [15]. This study aimed to evaluate and compare the wear resistance of acrylic resin, composite resin, and ceramic materials commonly used in removable dentures. It seeks to assess the extent of wear patterns and surface alterations exhibited by each material following simulated chewing cycles, thereby providing insights into their durability under realistic oral conditions. Additionally, this study endeavors to offer evidence-based recommendations for denture material selection, emphasizing the importance of wear resistance characteristics in enhancing denture longevity and patient satisfaction.

## Materials and Methods:

## Specimen selection and preparation:

Eighty-eight artificial tooth specimens were selected for this study, comprising three different materials commonly used in removable dentures: acrylic resin (n=30), composite resin (n=30), and ceramic (n=28). Specimens were obtained from reputable dental suppliers (DentsplySirona, IvoclarVivadent) to ensure consistency in the material quality and manufacturing standards. Standardized dimensions of 10 mm x 10 mm x 5 mm were used for all the specimens to minimize the variability in the surface area and volume.

## Wear testing protocol:

## Experimental setup:

A custom-designed wear-testing machine was employed to evaluate wear resistance. This machine was selected because of its ability to accurately simulate masticatory forces encountered in clinical settings. Each specimen was meticulously mounted onto a wear apparatus using a specialized fixture. This fixture ensured precise and uniform positioning of the specimens throughout the testing period, thereby minimizing the variability in load application and ensuring consistent wear testing conditions.

## Simulation parameters:

The wear-testing protocol was designed to replicate chewing cycles under controlled laboratory conditions, with the aim of assessing the wear resistance of acrylic resin, composite resin, and ceramic materials used in removable dentures.

[1] Load: A static load of 50 N was applied to each specimen during wear testing. This load intensity was selected to simulate the typical masticatory forces experienced by dentures during chewing in patients.

[2] Speed: The wear-testing machine was operated at a frequency of 60 cycles per minute. This frequency corresponds to the average chewing rate observed in individuals wearing dentures, ensuring that the simulated wear conditions closely mimicked real-world oral dynamics.

[3] Environmental Conditions: Tests were conducted under controlled environmental conditions, maintaining a constant temperature of 37°C. This temperature setting was chosen to simulate the physiological conditions of the oral cavity, ensuring that the wear characteristics of the materials were evaluated under clinically relevant temperatures, and to minimize the influence of external environmental factors on wear behaviour.

## Wear testing procedure:

Each specimen underwent a meticulous wear testing protocol designed to replicate the real-world conditions experienced by removable dentures. The procedure involved subjecting the specimens to 100,000 simulated chewing cycles to emulate the long-term wear that dentures endure in clinical settings.

## Monitoring and inspection:

Throughout the wear-testing duration, the specimens were inspected regularly under controlled lighting conditions. This periodic examination was crucial for researchers to closely monitor the progression of wear and meticulously record any visible changes in the surface characteristics of the materials. Researchers have documented various surface alterations, including micro fractures, abrasion patterns and changes in the surface roughness. These observations provide valuable insights into how materials respond to simulated chewing forces and wear conditions.

## Measurement of wear:

## Profilometric analysis:

After the completion of the wear-testing phase, a detailed quantitative assessment of the surface wear was conducted using a non-contact profilometer. This instrument facilitated three-dimensional surface scans of each specimen, allowing researchers to precisely measure wear depths and evaluate wear patterns across different surface areas. Specifically, measurements were taken from distinct regions of the specimens, including the mesial (toward the front), distal (toward the back), and occlusal (biting) surfaces. This comprehensive analysis helped characterize the spatial distribution of wear on the specimens and provided quantitative data essential for evaluating the performance of the materials under simulated chewing conditions.

## Data collection and analysis:

Wear depth measurements were recorded at multiple points on each specimen to ensure a comprehensive evaluation of wear distribution and magnitude. Data collected from profilometric scans were processed using specialized software (MountainsMap, Alicona Imaging GmbH) to calculate the mean wear depths and standard deviations for each material group (acrylic resin, composite resin and ceramic).

## Ethical considerations:

This study utilized commercially available dental materials and did not involve human or animal subjects, thereby exempting them from ethical approval. All the experimental procedures adhered to the ethical guidelines for scientific research and dental material testing, ensuring the integrity and reliability of the study outcomes.

## Statistical analysis:

Statistical analysis was performed using SPSS version 24.0 to determine the significant differences in the wear resistance among the three materials tested. The mean wear depth data were subjected to one-way analysis of variance (ANOVA) to assess overall differences between the material groups. Post-hoc Tukey tests were conducted for pairwise comparisons between materials to identify specific differences in wear resistance properties. A significance level of α=0.05 was used to establish statistical significance in wear performance between acrylic resin, composite resin and ceramic materials.

## Results:

The table presents the mean wear depths and standard deviations observed for the acrylic resin, composite resin and ceramic materials after subjecting them to 100,000 simulated chewing cycles. Acrylic resin exhibited the highest mean wear depth of 32.5 µm, with a standard deviation of 5.2 µm, indicating moderate wear characteristics. The composite resin demonstrated a lower mean wear depth of 24.8 µm (SD = 4.6 µm), suggesting improved wear resistance compared to acrylic resin. Ceramic displayed the lowest mean wear depth of 18.3 µm (SD = 3.9 µm), highlighting its superior resistance to wear under simulated chewing conditions.

Statistical analysis using one-way ANOVA revealed significant differences in wear resistance among the acrylic resin, composite resin and ceramic materials (F(2, 85) = 15.78, p < 0.001). Post-hoc Tukey tests further elucidated these differences: ceramic showed a significantly lower mean wear depth compared to both acrylic resin (p < 0.001) and composite resin (p = 0.004). Although the composite resin exhibited a lower wear depth than the acrylic resin, the difference was not significant (p = 0.123). These findings underscore ceramic as the most wear-resistant material tested, followed by composite resin and acrylic resin, aligning with the clinical expectations for durable denture materials.

The wear pattern analysis table provides insight into the distribution of wear across the mesial, distal and occlusal surfaces of the tested materials. For acrylic resin, wear depths were consistently higher across all surfaces: mesial (30.2 µm), distal (33.1 µm), and occlusal (35.0 µm) surfaces were consistently higher across all surfaces. Composite resin demonstrated intermediate wear characteristics with lower wear depths: mesial (22.5 µm), distal (25.4 µm), and occlusal (27.3 µm). In contrast, ceramic exhibited the least wear across all surfaces: mesial (16.7 µm), distal (17.9 µm), and occlusal (19.5 µm) surfaces exhibited the least wear across all surfaces. These results indicate that the ceramic maintains smoother surfaces with minimal wear, suggesting superior resistance to abrasive forces compared with acrylic and composite resins.

## Wear resistance of artificial teeth materials:

(Table 1 and Table 2) This study evaluated the wear resistance of three materials commonly used in removable dentures: acrylic resin, composite resin and ceramic. The mean wear depths (µm) and standard deviations were calculated based on profilometric analysis of specimens subjected to 100,000 simulated chewing cycles.

Statistical analysis using one-way ANOVA demonstrated significant differences in wear resistance among the acrylic resin, composite resin, and ceramic materials (F (2, 85) = 15.78, p < 0.001). Post-hoc Tukey tests indicated that the ceramic exhibited a significantly lower mean wear depth than acrylic resin (p < 0.001) and composite resin (p = 0.004). The composite resin also exhibited a lower wear depth than the acrylic resin, although the difference was not statistically significant (p = 0.123).

## Wear pattern analysis:

(Table 3) Detailed examination of wear patterns across specimens revealed consistent wear distributions on the mesial, distal and occlusal surfaces for all materials. The ceramic exhibited smoother wear surfaces with minimal abrasive wear compared to acrylic and composite resins, which displayed micro-cracking and surface roughness indicative of abrasive wear.

## Discussion:

## Wear Resistance and Clinical Implications

The results of this study demonstrated significant differences in the wear resistance among acrylic resin, composite resin and ceramic materials. Ceramic exhibited the lowest mean wear depth of 18.3 µm, followed by composite resin with 24.8 µm and acrylic resin with 32.5 µm. These findings align with previous research indicating the superior wear resistance of ceramics owing to their hardness and resistance to abrasive forces [16,17]. Despite its widespread use in denture fabrication and its ease of manipulation and cost-effectiveness, acrylic resin showed the highest wear depth among the materials tested. This can be attributed to the lower hardness and susceptibility of the acrylic resin to abrasive wear over time [4]. The wear patterns observed across the mesial, distal and occlusal surfaces further highlight the tendency of acrylic resin to develop microcracks and surface roughness, potentially affecting denture longevity and patient comfort [3]. Composite resin, a hybrid material combining a resin matrix and reinforcing fillers, demonstrated intermediate wear resistance compared with acrylic resin and ceramic. The wear characteristics of this material can vary depending on the filler content and bonding properties, thereby influencing its performance in denture applications [5,18]. The wear pattern analysis revealed smoother wear surfaces for the composite resin compared to the acrylic resin, suggesting enhanced resistance to abrasive wear despite having higher wear depths than ceramic [19].

## Factors influencing wear resistance:

Several factors influence the wear resistance of denture materials, including the material composition, hardness, surface finish and interaction with masticatory forces. Ceramic materials, typically composed of high-strength ceramics such as zirconia or alumina, exhibit superior mechanical properties and wear resistance owing to their crystalline structures and high hardness values [7]. These materials maintain smooth surface textures and resist abrasive wear, thereby contributing to improved durability in denture applications [20]. In contrast, acrylic resins, composed of polymethyl methacrylate (PMMA), offer favorable aesthetic properties and ease of processing, but are prone to wear and surface degradation over time [21]. The wear mechanism in acrylic resin involves micro fracture and abrasion, leading to increased roughness and potentially compromising denture fit and function [22]. Strategies to enhance wear resistance in acrylic resins include incorporating reinforcing agents or modifying polymerization techniques to improve material strength and longevity [11]. Composite resins in dentistry have evolved to offer a balance between aesthetic appeal and mechanical properties that are suitable for prosthetic applications. These materials combine a resin matrix with ceramic or glass fillers, providing improved wear resistance and durability compared with traditional acrylic resins [12]. However, composite resins may still exhibit wear characteristics influenced by the filler particle size, distribution, and bonding with the resin matrix, impacting their performance in denture prosthetics [13].

## Clinical implications and material selection:

The choice of denture material plays a critical role in determining the clinical outcomes, patient satisfaction and long-term denture performance. Ceramic materials have emerged as a preferred option for patients requiring durable and wear-resistant dentures, particularly in cases where longevity and functional integrity are paramount [14]. The superior wear resistance of ceramics ensures minimal surface wear and maintenance of occlusal stability over extended periods, contributing to improved patient comfort and a reduced need for denture adjustments [15]. Acrylic resins remain a viable choice for provisional or temporary dentures owing to their affordability and ease of modification. However, clinicians should consider their lower wear resistance and susceptibility to wear-related complications such as loss of occlusal morphology and decreased masticatory efficiency over time [23]. Regular maintenance and periodic replacement may be necessary to mitigate wear-related issues and ensure optimal denture function in acrylic resin-based prosthetics [24]. Composite resins offer a versatile alternative for denture fabrication, combining aesthetic appeal with enhanced mechanical properties compared to acrylic resins. The intermediate wear resistance of composite resins makes them suitable for patients seeking durable and aesthetically pleasing dentures without the high cost associated with ceramic materials [25]. Advances in composite resin technology continue to refine material formulations and processing techniques, enhance wear resistance and extend the lifespan of composite resin-based dentures in clinical practice [26].

## Limitations and future directions:

This study had several limitations that may influence the generalizability of the findings. The simulated wear-testing conditions, although designed to mimic clinical chewing cycles, may not fully replicate the complex oral environment and individual patient factors influencing denture wear [20]. Future research could explore additional variables, such as saliva composition, dietary habits and oral hygiene practices, to better understand the real-world performance of denture materials. Furthermore, the focus of this study on material wear resistance highlights the need for comprehensive clinical evaluations to assess other factors impacting denture longevity, including biocompatibility, tissue response and patient-specific considerations [21]. Longitudinal studies involving patient cohorts and clinical outcome assessments could provide valuable insights into the comparative effectiveness of different denture materials in the real-world settings [22].

In conclusion, this study contributes to the understanding of the wear resistance of artificial tooth materials for removable dentures. Ceramic is the most wear-resistant material among acrylic resins, composite resins and ceramics, demonstrating superior mechanical properties and durability under simulated chewing conditions.

## Figures and Tables

**Table 1:** Mean wear depths of artificial teeth materials

**Material**	**Mean Wear Depth (µm)**	**Standard Deviation (µm)**
Acrylic Resin	32.5	5.2
Composite Resin	24.8	4.6
Ceramic	18.3	3.9

**Table 2:** Results of statistical analysis (ANOVA)

**Comparison**	**F-value**	**p-value**
Acrylic Resin vs. Composite Resin	6.21	0.123
Acrylic Resin vs. Ceramic	18.57	< 0.001
Composite Resin vs. Ceramic	9.38	0.004

**Table 3:** Wear Pattern Analysis

**Surface**	**Acrylic Resin (µm)**	**Composite Resin (µm)**	**Ceramic(µm)**
Mesial	30.2	22.5	16.7
Distal	33.1	25.4	17.9
Occlusal	35	27.3	19.5
